# Exploring the Molecular Structure and Treatment Dynamics of Cellulose Fibres with Photoacoustic and Reversed Double-Beam Spectroscopy

**DOI:** 10.3390/polym16233419

**Published:** 2024-12-05

**Authors:** Levente Csóka, Worakan Csoka, Ella Tirronen, Ekaterina Nikolskaya, Yrjö Hiltunen, Bunsho Ohtani

**Affiliations:** 1 Faculty of Informatics, ELTE Eötvös Loránd University, 1053 Budapest, Hungary; 2 Fiber Laboratory, South-Eastern Finland University of Applied Sciences, 57200 Savonlinna, Finland; cs.worakan@celltech-paper.hu (W.C.); ella.tirronen@xamk.fi (E.T.); ekaterina.nikolskaya@xamk.fi (E.N.); yrjo.hiltunen@xamk.fi (Y.H.); 3 Nonprofitable Organization Touche NPO, 1-6-414, North 4, West 14, Sapporo 060-0004, Japan; bunshoohtani@gmail.com

**Keywords:** cellulose spectroscopy, light absorption, crystallinity, reversed double-beam photoacoustic spectroscopy, molecular dynamics

## Abstract

In this study, we explored the structural and chemical modifications of cellulose fibres subjected to chemical and mechanical treatments through an innovative analytical approach. We employed photoacoustic spectroscopy (PAS) and reversed double-beam photoacoustic spectroscopy (RDB-PAS) to examine the morphological changes and the chemical integrity of the treated fibres. The methodology provided enhanced sensitivity and specificity in detecting subtle alterations in the treated cellulose structure. Additionally, we applied Coifman wavelet transformation to the PAS signals, which facilitated a refined analysis of the spectral features indicative of chemical and mechanical modifications at a molecular level. This advanced signal processing technique allowed for a detailed decomposition of the PAS signals, revealing hidden characteristics that are typically overshadowed in raw data analyses. Further, we utilised the concept of energy trap distribution to interpret the wavelet-transformed data, providing insights into the distribution and density of energy states within the fibres. Our results indicated significant differences in the energy trap spectra between untreated and treated fibres, reflecting the impact of chemical and mechanical treatments on the fibre’s physical properties. The combination of these sophisticated analytical techniques elucidated the complex interplay between mechanical and chemical treatments and their effects on the structural integrity and chemical composition of cellulose fibres.

## 1. Introduction

Photoacoustic spectroscopy [1] (PAS) and reversed double-beam photoacoustic spectroscopy [2] (RDB-PAS) are two powerful techniques used in spectroscopic analysis to investigate the properties of materials. Photoacoustic spectroscopy (PAS) operates by inducing the absorption of modulated light energy by a sample. When the light is absorbed, it generates localised heating within the sample, leading to thermal expansion and the emission of acoustic waves [3]. The intensity of these acoustic waves is directly proportional to the concentration of the absorbing species within the sample under the simplest assumption. By measuring the intensity of the generated acoustic waves, PAS provides quantitative information about the concentration of the target species, enabling precise analysis of samples in various applications [4].

In contrast, RDB-PAS is a variation of newly developed double-beam spectroscopy where the reference beam is intensity-modulated. This technique offers advantages such as improved signal-to-noise ratio and enhanced sensitivity compared to conventional methods. By modulating the reference beam, RDB-PAS discriminates against common sources of noise and is particularly useful for studying weak signals and low concentrations in complex samples.

In materials science, PAS and RDB-PAS are employed for analysing thin films, nanomaterials, polymers, and semiconductors. It is important to emphasise that PAS and RDB-PAS are techniques to measure the photoabsorption-induced acoustic waves and not the intensity reduced by photoabsorption of the sample.

Cellulose, a polysaccharide composed of carbon, hydrogen, and oxygen, exhibits distinct light-absorbing properties influenced by its chemical structure, bonding configuration, and crystallinity [5]. Cellulose is characterised by both crystalline and amorphous domains, each contributing differently to its spectroscopic behaviour [6]. Cellulose exhibits inherent or induced electron traps or defects, which influence its behaviour under various conditions.

The light-absorbing capacity of cellulose is dependent on its molecular structure and influenced by charge densities [7,8], electrostatic interactions, and surface interactions, characterised by dispersive and polar forces [9,10]. Single carbon–carbon bonds within the cellulose backbone predominantly absorb ultraviolet (UV) radiation with wavelengths shorter than 200 nm. Additionally, carbon–oxygen bonds, such as those found in hydroxyl groups (-OH), contribute to absorption in the UV [11] and visible regions. Cellulose’s crystalline regions, characterised by well-ordered hydrogen bonding networks, exhibit specific absorption bands due to their electronic structure [12], whereas amorphous regions display broader absorption features.

Oxygen’s light absorption in cellulose is influenced by its bonding configuration. Single oxygen–oxygen bonds in cellulose generally do not absorb light in the visible or UV range, but may contribute to absorption in the infrared region due to vibrational modes. Oxygen-containing functional groups, such as hydroxyl (OH) groups, contribute to absorption in the UV and visible regions [13].

Hydrogen’s light absorption properties in cellulose are primarily determined by its chemical environment [14]. Hydrogen atoms within cellulose, particularly those involved in hydrogen bonding interactions, can contribute to absorption in the UV and visible regions.

Cellulose, being fundamentally an insulator, requires high-energy photons for electronic transitions to occur. In our earlier study [15], electromagnetic excitation processes revealed electronic vibration bands (phonons) and excitons, suggesting photonic or vibrational behaviours that are more complex than simple dielectric behaviour. These phenomena could be related to localised electronic states, defects, or interactions with molecular vibrations, but they do not imply the presence of free electron movement within the cellulose molecule.

The aim of this study was to deepen our understanding of the light-absorbing properties of cellulose under various chemical and mechanical treatments, which is crucial for interpreting photoacoustic spectroscopic data and could enable the design of cellulose-based materials with tailored optical characteristics. By investigating how different treatments affect cellulose fibres’ interaction with electromagnetic radiation, we hope to reveal new insights into the molecular structure and surface dynamics of this abundant biopolymer. These findings could bridge knowledge gaps in cellulose chemistry, offering potential applications across materials science, biotechnology, and environmental science.

## 2. Materials and Methods

### 2.1. Materials

Whatman filter paper (WFP) crafted from cotton linters was sourced through GE Healthcare. Additionally, softwood (SW; pine) and hardwood pulp (HW; birch) were included in the tests.

### 2.2. Methods

#### 2.2.1. Chemical Treatment

The WFP samples underwent a chemical hydrolysis treatment using HCl. This procedure involved the use of HCl vapor within a sealed desiccator that housed a petri dish filled with HCl solution. To replace the internal air of the desiccator completely with an HCl atmosphere, the valve of the desiccator was left open for 48 h. After this period, filter paper was placed inside the desiccator for hydrolysis durations of 2, 4, 6, and 8 h. The designations such as WFP_C, WFP_2h, WFP_4h, WFP_6h, WFP_8h correspond to specific treatment conditions applied to Whatman filter paper. Post-hydrolysis, the filter paper was thoroughly washed in water for 24 h to eliminate any residual HCl. The filter paper was subsequently dried at 60 °C in a drying oven.

#### 2.2.2. Mechanical Treatment

The SW and HW samples underwent mechanical processing using an AB Lorentzen & Wettre laboratory valley beater, with the duration of beating varied among the samples. The designations such as HW_C, HW_20, HW_40, HW_60, SW_0, SW_30, SW_60, and SW_90 correspond to specific treatment conditions applied to the wood fibre samples. The number at the end of each sample specifies the duration of the beating in minutes.

#### 2.2.3. Photoacoustic Spectroscopy

PAS (photoacoustic spectroscopy) is effective in analysing the structural and chemical modifications in cellulose fibres by detecting the absorption of light and the resulting thermal waves, providing insights into the molecular composition and changes within the material.

A custom-built photoacoustic (PA) cell was utilised, featuring an aluminium body with an internal volume of approximately 0.5 cm^3^, a micro-electromechanical system (MEMS) microphone module (SparkFun MEMS Microphone Breakout, INMP401 (ADMP401), Niwot, CO, USA), and a quartz-glass window that allowed transparency across the 250–1000 nm measurement range. The cell was temperature-stabilised at 298.0 K using a block heating–cooling bath (AS ONE Dry Block Bath MyBL-100C, manufactured by AS ONE Corporation, Osaka, Japan) to minimise temperature fluctuations during measurements. A cellulose pulp sample was placed inside the cell, and PA spectra were recorded at room temperature under a nitrogen atmosphere. Pulp samples were cut into circular shapes with a diameter of 10 mm and an approximate thickness of 0.5 mm. Monochromatic light (~0.2 mW/cm^2^) was provided by a monochromator (Bunkokeiki, Tokyo, Japan). The PAS signal detected by the MEMS microphone module within the cell was amplified and analysed using a digital lock-in amplifier (NF LI5640). To account for variations in light intensity across different wavelengths, the PAS signal was normalised using carbon black (graphite) powder as a reference.

#### 2.2.4. Reversed Double-Beam Photoacoustic Spectroscopy Measurement

RDB-PAS (reversed double-beam photoacoustic spectroscopy) enhances the sensitivity and specificity of structural and chemical analysis by using a reversed double-beam setup, allowing for the differentiation of subtle changes in the absorption characteristics of treated cellulose fibres. Unlike other spectroscopic techniques, which primarily measure light absorption or transmission, PAS provides a non-destructive approach that translates photoabsorption events directly into acoustic signals, enhancing sensitivity to molecular-level changes within the cellulose structure. The RDB-PAS measurement technique was adapted from a previous methodology with certain specific alterations [15]. In these measurements, the focus was on the detection of acoustic waves generated by the absorption of photons. We employed a custom-built photoacoustic (PA) cell (used in PA measurement), which was outfitted with a MEMS microphone module and a quartz-glass window allowing transparency from 250 to 1000 nm, to reduce external environmental impacts. The RDB-PAS analyses utilised monochromatic light scanning from 600 to 250 nm and a modulated near-infrared (940 nm) LED at 35 Hz, roughly 8 mm in diameter. The RDB-PAS signals, indicative of the total density of trapped electrons, were captured directly without any signal normalisation. To mitigate potential influences of environmental variables on PAS and RDB-PAS measurements, carefully controlled experimental conditions were maintained. Cellulose samples were thoroughly dried before photoacoustic measurements, and analyses were conducted under static conditions at room temperature. The ambient conditions for these measurements consisted of static, methanol-saturated nitrogen. Before the RDB-PAS measurements commenced, the cell was flushed with nitrogen gas infused with methanol vapor for 10 min at a flow rate of 30 mL/min.

The experimental setup for RDB-PAS involved the alternating use of modulated and continuous light beams. This arrangement allowed the continuous light to excite electrons into electron traps (ETs), while the modulated light simultaneously monitored the photoabsorption by these electron-filled traps, thus documenting the spectrum of photoinduced trap-filling. The cell was preconditioned with methanol-saturated nitrogen gas, which served to irreversibly immobilise positive holes, thereby preventing the annihilation of electrons once trapped, through their reactions with these positive holes.

For a brief interpretation and details of the PAS and RDB-PAS measurements, see the Supplementary Materials.

The results presented are the average of three measurements, with a reported error of 3–5%.

#### 2.2.5. Signal Processing

The photoacoustic spectra were deconvoluted using PeakLab software, version 1.05.07 and discrete wavelet transformation was performed using IgorPro software, version 9.0.5.1 (WaveMetrics).

#### 2.2.6. Coifman Wavelet Analysis

Coifman wavelet analysis (named after Ronald R. Coifman) provides a multiresolution analysis to decompose photoacoustic signals into different frequency components at various resolutions [16]. The localisation of frequency components within the wavelength domain allows for a more detailed, scale-sensitive analysis of spectral data, enhancing the detection of subtle, treatment-induced molecular changes in cellulose. Coifman wavelets were generated algorithmically due to their complex nature, and 2 vanishing moments to detect polynomial trends in the photoacoustic signal were selected. This means that the wavelet is orthogonal to constant (degree 0, wavelet has mean of zero) and linear (degree 1, wavelet is orthogonal to linear trends in the photoacoustic signal) functions, ignoring these trends and enhancing higher-order features [17]. Discrete wavelet transformation (DWT) helped in identifying and separating specific surface chemistry features from the photoacoustic signals of cellulose fibres with varying spatial frequencies. Coifman DWT effectively denoised the photoacoustic signal and preserved the structural features of cellulose for accurate interpretation and analysis. The localisation of absorbers or sources of the photoacoustic signal within cellulose fibres is essential to understand the structural properties.

## 3. Results

### 3.1. Chemical Treatment

**Figure 1:**
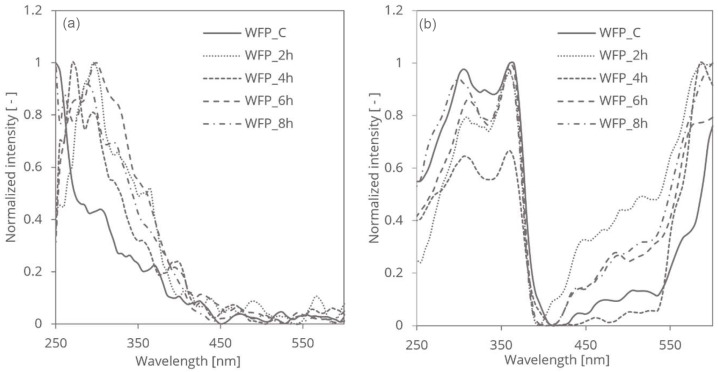
Photoacoustic spectroscopy (**a**) and reversed double-beam photoacoustic (**b**) spectra of WFP samples.

The treatment of WFP cellulose with HCl vapor introduced chemical modifications that significantly influenced its light-absorbing properties and altered its spectroscopic behaviour and optical characteristics (Figure 1).

This chemical modification is consistent with findings from studies on various types of treated cellulose fibres such as textile [18] or dialdehyde cellulose nanofibrils (DACNFs) derived from cotton and wood fibres, where periodate oxidation was shown to disrupt crystalline regions and alter optical properties [19]. Similarly, UV-visible diffuse reflectance spectroscopy has been employed to analyse lignocellulosic materials, revealing how chemical treatments, like HCl vapor, can modify UV absorption bands, particularly in regions associated with residual lignin and other impurities [20].

HCl vapor treatment chemically modifies cellulose by selectively hydrolysing glycosidic linkages, cleaving cellulose chains in the disordered regions, and introducing functional groups such as hydroxyl (OH) and carboxyl (COOH) groups onto the cellulose backbone. Such functionalisation is known to enhance or diminish the light-absorbing properties depending on the specific treatment and the type of cellulose involved, as seen in mechanically treated and UV-treated regenerated cellulose fibres, where changes in surface energy and fibre–matrix adhesion significantly impacted optical behaviour [21].

Figure 1 illustrates the distinct results obtained from photoacoustic spectroscopy (PAS) and reversed double-beam photoacoustic spectroscopy (RDB-PAS) for the same cellulose samples. The significant differences in the spectra arise from the unique aspects each technique measures.

Photoacoustic spectroscopy (PAS) primarily detects the absorption-induced acoustic signals, which reflect changes in the sample’s electronic and vibrational transitions. In the case of cellulose fibres, PAS spectra are sensitive to variations in the disordered regions and overall molecular interactions, capturing shifts and broadening of absorption bands that correlate with structural changes such as reductions in disordered regions, increases in crystallinity, and modifications in molecular weight and degree of polymerisation.

A similar pattern in PAS spectra can be found in the literature for microcrystalline cellulose, hemicelluloses, and lignin [22].

The spectral shifts are similar to those observed in UV spectroscopy studies of cellulose, where chemical treatments lead to broadened absorption bands in the UV range, particularly between 250–400 nm, corresponding to changes in molecular structure [23,24].

Conversely, reversed double-beam photoacoustic spectroscopy (RDB-PAS) employs a different approach by utilizing two beams of light to enhance sensitivity and minimise noise. This technique measures the relative changes in signal intensity, providing insights into the material’s structural modifications, such as changes in the crystallinity and the impact of the treatment on the cellulose fibres. Studies on mechanically treated cellulose also demonstrate how techniques like RDB-PAS can be used to assess changes in surface area and light-scattering properties, especially in fibres with increased surface roughness due to mechanical grinding [21].

The RDB-PAS spectra therefore focus on detecting intensity variations rather than peak shifts or broadening, which explains the discrepancies observed between Figure 1a and Figure 1b.

The divergence in the spectra between PAS and RDB-PAS underscores the complementary nature of these methods. PAS provides detailed insights into electronic and vibrational transitions, highlighting subtle changes in the chemical structure and environment of the cellulose fibres. In contrast, RDB-PAS emphasizes relative intensity variations, which can offer additional perspectives on the sample’s optical properties. Together, these techniques provide a more comprehensive understanding of the modifications in cellulose fibres resulting from the applied treatments.

This complementary analysis is also reflected in studies that compare PAS and RDB-PAS for cellulose materials subjected to different treatments, underscoring how both techniques provide critical insights into the effects of chemical and mechanical modifications on optical properties [25].

The introduction of functional groups did not introduce any surface charges or disruptions in cellulose structure caused by HCl vapor treatment; however, they significantly impacted its light-absorbing properties. In Figure 1a, the normalised photoacoustic spectroscopy (PAS) spectra exhibit shifts in the main peaks towards higher UV wavelengths over different treatment times. Specifically, the spectra corresponding to 2, 4, 6, and 8 h of HCl vapor treatment show varying degrees of peak shifts and broadening. These changes reflect similar phenomena observed in studies where chemical treatments induced crystallisation and reduced disordered regions, thereby affecting the UV absorption spectra [26]. Initially, as the treatment progresses from 2 to 4 h, there is an observable shift and broadening of the absorption bands, indicating changes in the electronic and vibrational transitions within the disordered regions of cellulose. This trend becomes more pronounced at 6 h, where the crystallisation process becomes more significant, leading to further shifts and broadenings. By 8 h, the spectra stabilise, showing less pronounced changes in peak positions, but continuing to reflect the evolved structural state of the cellulose.

In contrast, the RDB-PAS measurements shown in Figure 1b maintain the peak positions consistent with those observed in PAS, but with variations primarily in intensity. The highest normalized intensity difference is observed in the sample treated for 4 h, within the 250–370 nm range, indicating significant changes in the material’s structure at this stage.

HCl vapor treatment promotes the reduction of amorphous regions in cellulose, thereby increasing its crystallinity ratio, without altering the conformational integrity of the crystalline regions. Additionally, it halts the hydrolysis of glycosidic linkages at these crystalline regions. This aligns with findings demonstrating that chemical treatments can stabilize or enhance the crystallinity of cellulose fibres, leading to consistent spectral peaks despite variations in intensity [20].

**Figure 2:**
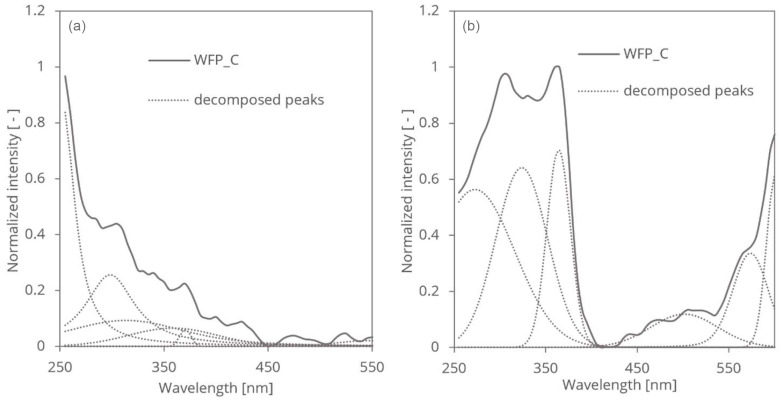
Decomposed PAS (**a**) and RDB-PAS (**b**) spectra of WFP samples.

Figure 2 presents the PAS and RDB-PAS spectra, which provide complementary insights into the changes induced by treatment. PAS spectra reveal shifts and broadening of absorption bands within the UV range (250–400 nm), indicating changes in the electronic and vibrational states associated with disordered regions of cellulose. In contrast, RDB-PAS spectra emphasize relative intensity variations, reflecting structural and compositional alterations in the treated samples.

For the WFP samples, the areas of the decomposed Voigt-type peaks in the PAS spectra initially increased with reduced UV energy up to the WFP_4h sample, followed by a decrease. Conversely, in the RDB-PAS spectra, the decomposed peak areas decreased until WFP_4h and then increased.

For SW samples, the areas of the PAS decomposed peaks decreased, then increased, and subsequently decreased again as treatment time progressed. The RDB-PAS spectra showed a consistent decreasing trend in the peak areas.

For HW samples, the PAS decomposed peak areas increased steadily with treatment time, while the RDB-PAS spectra exhibited a decrease in peak areas up to 40 min of treatment, followed by an increase.

These observations highlight the complementary nature of PAS and RDB-PAS in capturing the effects of treatment on cellulose fibres, with PAS focusing on electronic and vibrational state changes and RDB-PAS emphasizing relative intensity changes.

Furthermore, the introduction of hydroxyl and carboxyl groups onto the cellulose backbone altered its chemical reactivity and interaction with incident light. Additionally, alterations in surface chemistry and morphology induced by HCl vapor treatment can influence the refractive index and light-scattering properties of cellulose materials [27].

This is evident in other studies, where treatments led to changes in UV-visible spectra due to the introduction of such functional groups and their impact on the material’s optical properties [25].

### 3.2. Mechanical Treatment

The mechanical treatment of SW and HW cellulose fibres plays a crucial role in altering their light-absorbing properties, influencing their spectroscopic behaviour and optical characteristics. Mechanical treatments, such as grinding, have been shown to significantly modify the structural and optical properties of cellulose fibres, as highlighted in studies involving mechanical fibrillation and UV treatments [21]. Cellulose, a biopolymer composed of carbon, hydrogen, and oxygen, exhibits a complex molecular structure consisting of both crystalline and amorphous domains. These structural features contribute differently to the light absorption of cellulose. The mechanical impact is assessed through PAS and RDB-PAS techniques, each providing distinct insights.

**Figure 3:**
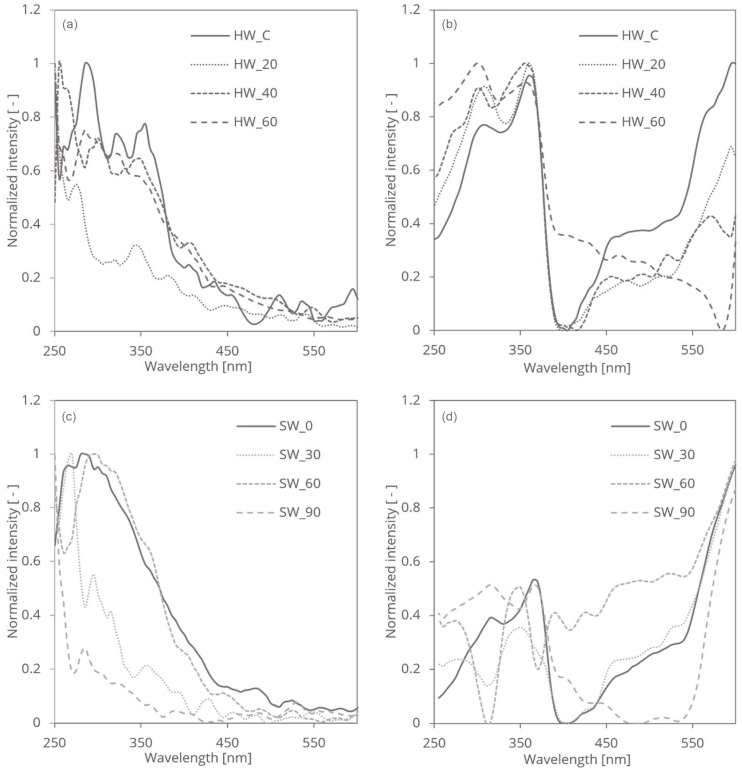
Photoacoustic spectroscopy (PAS) spectra of HW (**a**) and SW (**c**) samples and reversed double-beam photoacoustic spectroscopy (RDB-PAS) spectra of HW (**b**) and SW (**d**) samples.

Mechanical treatment, such as grinding, induces physical changes in cellulose structure, resulting in modifications to its crystallinity, particle size, and surface morphology. Specifically, mechanical forces applied during treatment can disrupt hydrogen bonding interactions and break down cellulose aggregates, leading to the fragmentation of cellulose fibres and the generation of smaller particles with increased surface area. These effects are particularly evident in studies that demonstrate how mechanical grinding enhances fibrillation and alters the UV absorption characteristics of cellulose fibres, especially in terms of surface roughness and light-scattering properties [26]. The mechanical treatment resulted in a progressive increase in fibrillation for softwood (SW) fibres as the treatment duration extended, while hardwood (HW) fibres exhibited an initial decrease in fibrillation followed by a subsequent increase. The mechanical grinding process enhanced the surface area of the fibres, as evidenced by the observed increase in fibrillation, measured by a demo version of a Valmet fractionator. Photoacoustic spectroscopy (PAS) measures the absorption-induced acoustic signals in different cellulose fibres, reflecting changes in the material’s electronic and vibrational transitions. PAS detects shifts and broadening of absorption bands that result from mechanical treatment-induced structural changes, such as reductions in crystallinity and the formation of defects or disordered regions. These spectral changes provide information about how the mechanical treatment affects the internal structure of cellulose, including the disruption of hydrogen bonding and the breakdown of cellulose aggregates (Figure 3a,c).

Reversed double-beam photoacoustic spectroscopy, on the other hand, focuses on detecting relative changes in signal intensity. This technique is sensitive to modifications in the surface area and morphology of cellulose particles. Such sensitivity is crucial in distinguishing the effects of different mechanical treatments, as seen in studies where RDB-PAS was employed to analyse the surface energy changes in UV-treated fibres [21]. For cellulose fibres subjected to mechanical grinding, RDB-PAS revealed how the treatment influenced the light-scattering and absorption properties due to changes in particle size and surface roughness. Enhanced fibrillation and increased surface area, as observed in both SW and HW fibres, led to more pronounced light scattering and absorption. RDB-PAS thus highlights the effects of mechanical treatment on the dispersion and interaction of cellulose particles with incident light (Figure 3b,d). For HW fibres, fibrillation initially decreased after 20 min of grinding compared to its starting value (1.5%), but then increased, reaching approximately 3.5% after 60 min of grinding. In contrast, SW fibres exhibited a gradual increase in fibrillation, achieving a 4.3% increase with extended treatment time. Notably, both SW and HW fibres reached a 2% relative fibrillation increase after approximately 60 min of treatment, suggesting that their response to mechanical treatment is relatively similar. This similarity makes it challenging to track changes using PAS and RDB-PAS measurements.

These structural changes correlate with trends observed in the RDB-PAS spectra, which emphasize the effects of mechanical treatment on the dispersion and interaction of cellulose particles with incident light. Specifically, the spectra exhibit changes in intensity and signal broadening, particularly within the UV region (250–400 nm). These effects are likely tied to the increased surface area and its influence on light scattering and absorption properties.

While this study highlights the observable trends in the spectra and their connection to fibrillation, a more detailed investigation into the precise relationship between surface area changes and spectral features (e.g., broadening, signal shifts, or the appearance of new signals) remains an area for future research.

In summary, PAS provides insights into the structural changes and alterations in light absorption within the cellulose fibres, while RDB-PAS emphasises how these structural modifications impact light-scattering and absorption properties. Both techniques together offer a comprehensive understanding of the effects of mechanical treatment on different cellulose fibres. This comprehensive approach aligns with recent research on the impact of mechanical treatments on cellulose’s optical properties, underscoring the importance of analysing both electronic and structural modifications [23].

Overall, the mechanical treatment of cellulose has profound effects on its light-absorbing properties, leading to alterations in its spectroscopic behaviour and optical characteristics. Understanding these effects is essential, as highlighted by multiple studies, for optimizing the processing and utilisation of cellulose-based materials in various applications, including papermaking, biomaterials, and renewable energy technologies [25].

### 3.3. Coifman Discrete Wavelength

The wavelet analysis of untreated and treated cellulose fibres yielded characteristic wavelet coefficient distributions in the wavelength domain. The resulting coefficients displayed peaks and localised features at distinct wavelength positions, revealing surface structure variations within the cellulose fibres (Table 1).

**Figure 4:**
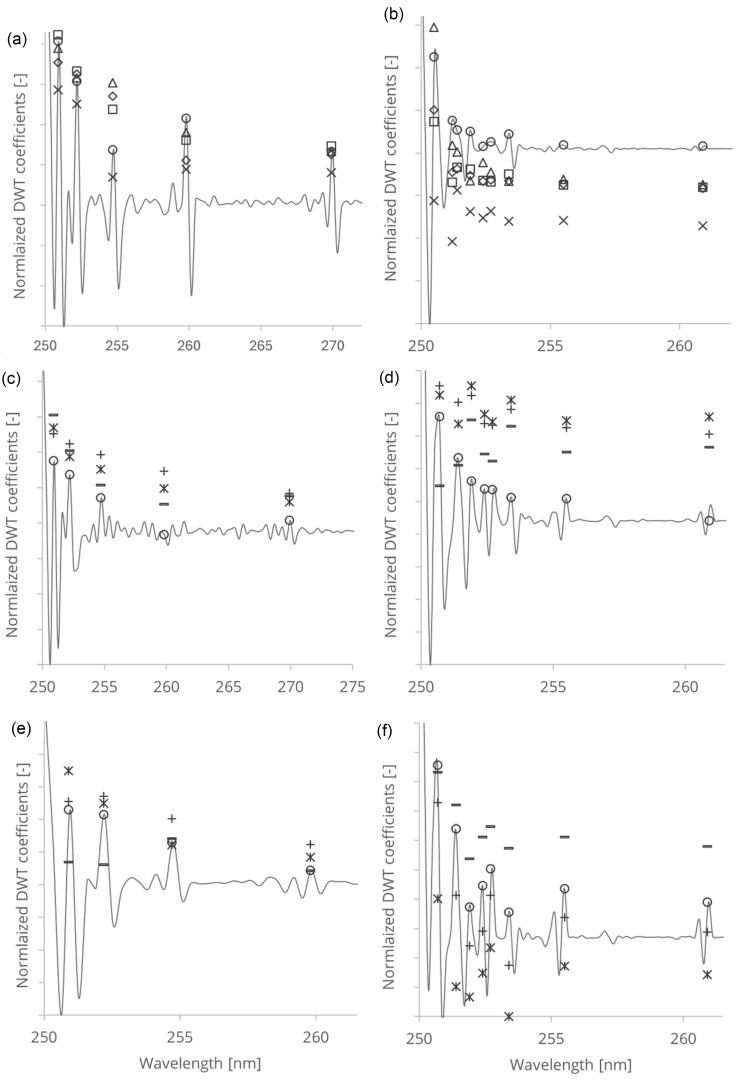
DWT of photoacoustic spectroscopy spectra of WFP (**a**), HW (**c**) SW, (**e**) and DWT of reversed double-beam photoacoustic spectroscopy spectra of WFP (**b**), HW (**d**), and SW (**f**) samples. The symbols indicate: o control, × 2 h, △4 h, ☐6 h,  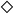  8 h, − DWT of control WFP; o  control, + 20 min, ∗ 40 min, − 60 min, – DWT of control HW; o control, + 30 min, ∗ 60 min, − 90 min, – DWT of control SW.

To quantify and visualise the changes induced by treatment, the wavelet coefficients of the treated samples were normalised (Figure 4). This approach allowed us to identify regions of increased or decreased absorbance, thereby highlighting the spectral areas most affected by the chemical and mechanical treatment. The resulting coefficient distributions provided a clear representation of structural modifications in cellulose over time, with discernible patterns that varied with treatment duration. This detailed mapping of coefficients enabled an understanding of how specific treatments altered the fibres’ absorbance characteristics.

The peaks in the wavelet coefficients correspond to the absorption of -OH groups within the PAS and RDB-PAS signals, localised at specific wavelength. The exponential decrease in magnitude and the position of the peaks provide insight into the strength and distribution of -OH group conformations in the PAS and RDB-PAS spectra. The observed decrease in peak intensity from UV to visible wavelengths indicates differences in the absorption characteristics of cellulose fibres across the spectrum (Figure 4).

Surface -OH group on cellulose exhibit stronger absorption in the UV range than in the visible range, resulting in an elevated photoacoustic response. Variations in the distribution and density of surface -OH groups contribute to differences in photoacoustic response at different wavelengths. Crystalline regions of cellulose fibres show higher UV absorbance than non-crystalline areas, likely due to electronic transitions or resonance effects within the ordered molecular lattice. This results in a more pronounced photoacoustic signal in the UV domain. The high ordering of -OH groups in crystalline cellulose regions contributes to the structural integrity and stability of the fibres, enhancing photoacoustic wave generation and creating a stronger response in the Coifman wavelet spectrum.

**Figure 5:**
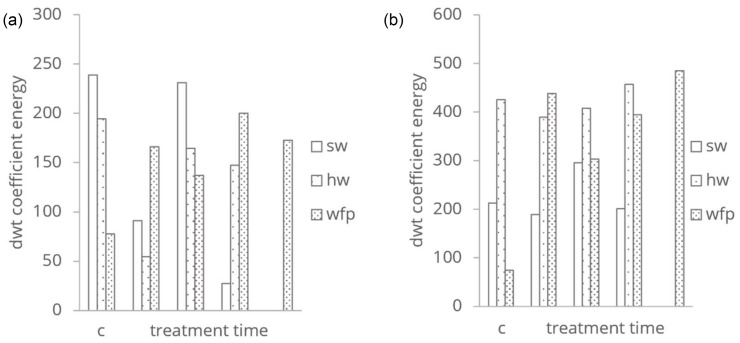
Integrated DWT coefficients of (**a**) PAS and (**b**) RDB-PAS spectra energy distribution with different treatment types.

To clarify the impact of mechanical treatment on softwood (SW) and hardwood (HW), we quantified the wavelet energy in the PAS and RDB-PAS spectra using the Coifman Discrete Wavelet Transform (DWT). By summing the squared coefficients, we generated an indicative measure of the signal’s energy distribution across frequencies and wavelengths, referred to as wavelet energy, which is shown in Figure 5. This energy measure reflects the total signal variance attributable to chemical and mechanical treatments. For both SW and HW cellulose fibres, the integrated DWT coefficients first decrease relative to the control, then increase, and eventually decrease again. This behaviour aligns with the known effects of mechanical treatment, which typically reduces crystallinity and increases molecular bonding due to enhanced fibre surface area [28]. Additionally, the different composition of SW and HW fibres might contribute to the observed differences in treatment response. Hardwood fibres, which contain more vessel elements compared to softwood, may exhibit unique mechanical and chemical behaviour under treatment, potentially leading to differences in the way they respond to the treatments and the resulting structural changes [28]. For chemically treated wood-free pulp (WFP) cellulose fibres, the integrated DWT coefficients initially rise, then decline, and increase with extended treatment time. During the early stages of HCl vapor hydrolysis, hydrogen chloride interacts with the cellulose surface, modifying its roughness and reducing amorphous regions, which may enhance the photoacoustic response.

After 4 h of treatment, energy levels decreased, though they remained above control levels, indicating progressive chemical modification where structural and compositional changes began to stabilise, optimizing the cellulose’s interaction with photoacoustic waves. By 6 h, the DWT coefficients showed a more complex pattern, suggesting a secondary phase of changes, such as crystallisation or other structural alterations, which further enhances the fibre’s UV absorption and photoacoustic response. Prolonged exposure to HCl vapor (8 h) leads to excessive cellulose degradation, reducing its heterogeneity and thereby diminishing its capacity to generate a strong photoacoustic signal. This excessive degradation results in a more uniform structure, with reduced heterogeneity contributing less to the photoacoustic response.

### 3.4. Electron Trap Distribution

The formation of electron traps on the cellulose surface involves complex interactions at the molecular level, leading to the creation of localised energy states that can capture and hold electrons temporarily [15]. These traps are defects or irregularities within the cellulose structure that have distinct electronic properties.

The mechanisms leading to the formation of these traps under the HCl vapor and mechanical grinding processing included the protonation of hydroxyl groups and cleavage of glycosidic bonds in the cellulose chain and mechanical disruption in crystalline structure creating amorphous areas in the cellulose chain, respectively.

**Figure 6:**
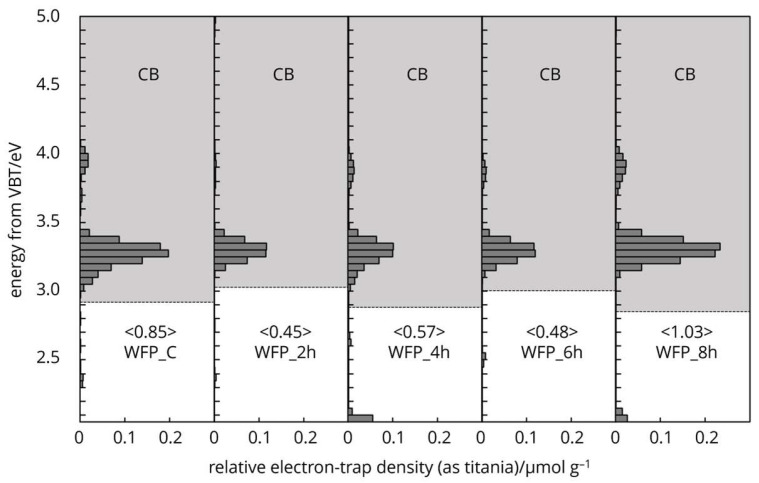
Electron trap distribution of HCl vapor-hydrolysed cellulose samples.

**Figure 7:**
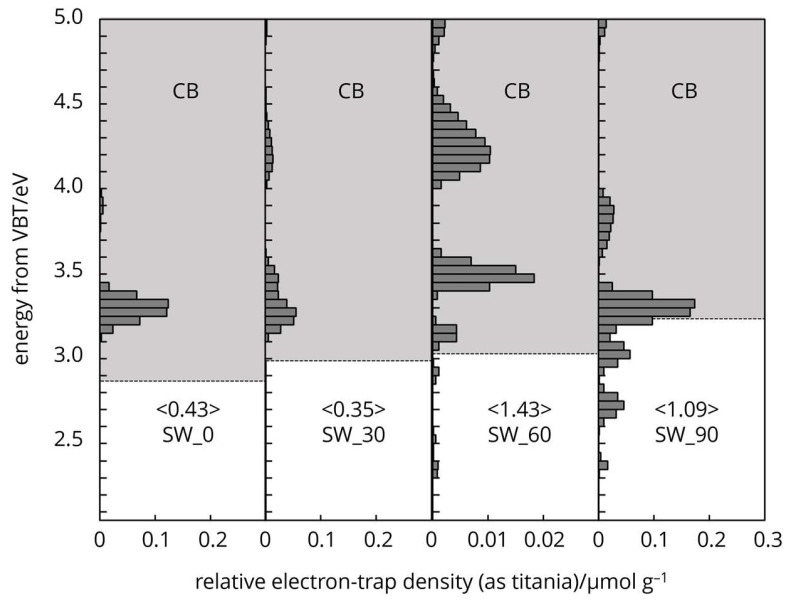
Electron trap distribution of mechanically treated SW cellulose samples.

**Figure 8:**
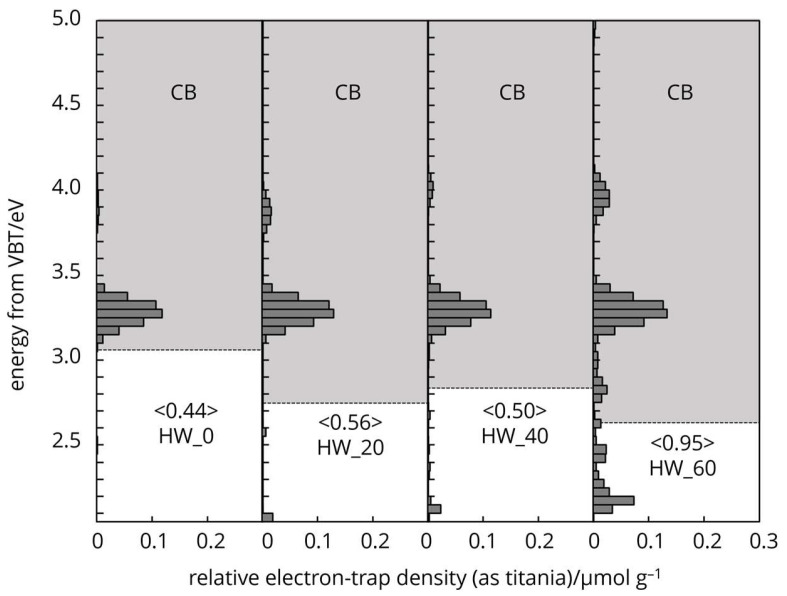
Electron trap distribution of mechanically treated HW cellulose samples.

Cellulose naturally possesses intrinsic-type electron traps, and the distribution of these traps in the energy levels spectra is unique (Figure 6, Figure 7 and Figure 8). The characteristic features arise from the two different crystallography phase structures and from imperfections or defects within the crystal lattice.

The extrinsic type of electron traps arose from the HCl vapor chemical and mechanical grinding processes.

The energy levels of the electron traps in differently processed cellulose fibres were below the conduction band overall, from 4.7 to 2.8 eV. This range indicates a broad distribution of trap depth. The energy distribution of electron traps centred around 4.3 eV (Figure 7) held the electrons tightly (deep traps), which were located further from the bands, effectively immobilizing them and influencing the non-linear dielectric properties (Salama 2004), storage of charge, and electrical insulation stability of cellulose [29,30,31]. Electron trap distribution around 3.2 eV (Figure 6, Figure 7 and Figure 8) loosely held electrons (shallow traps) and was located closer to the conduction band. The latter can easily release electrons back to the band, contributing to photoconductivity or slight increases in conductivity under illumination [32,33,34,35].

The presence of the characteristic electron trap distribution on the cellulose surface affects dielectric behaviour and how the charges move in non-radiative recombination centres and responses to external electric fields.

The protonation of hydroxyl groups and the potential cleavage of glycosidic bonds by HCl vapor treatment create new radical sites or carbocations for electron trapping [36]. The energy levels of the electron traps in chemically processed cellulose fibres were below the conduction band (grey area) of 4.1–3.0 eV (Figure 6). The deep electron trap band distribution of WFP type cellulose was located further toward the conduction band, centred at 3.9 eV in the control sample. As the HCl vapor hydrolysis time increased to 6 h, the deep electron bands moved to a lower energy range. As crystallisation occurred at 8 h treatment, the deep electron bands moved back to a higher energy level and at the same time increased in intensity. The shallow traps’ centre was not changed; however, the intensity increased at 8 h treatment and exceeded the control sample. The degree of polymerisation was reduced by 85% after 8 h treatment [37,38,39], resulting in higher crystallinity and thermally more stable cellulose material.

Mechanical grinding imposes physical changes, generates mechanical stress on the cell wall, leading to the disruption of the crystalline structure and the creation of amorphous regions. The physical distortion and creation of structural defects, such as dislocations in the cellulose fibres can lead to the formation of electron traps. The physical irregularities enhance cellulose ability to interact with electrons, facilitating the trapping mechanism. These defects disturb the periodic potential of the crystalline lattice, creating localised energy levels within the band gap, where electrons can be trapped (Figure 7 and Figure 8).

Given that the molecular changes induced by mechanical and chemical treatments are fundamentally distinct, their effects do not exhibit synergistic interactions. However, the potential for a combined treatment approach integrating both mechanical and chemical processes warrants further investigation. The PAS and RDB-PAS techniques offer valuable insights into how such combined treatments may influence the structural properties of cellulose, as well as the distribution of energy traps. This combined approach could facilitate a deeper understanding of the molecular modifications resulting from these treatments and their impact on cellulose’s physicochemical behaviour.

## 4. Conclusions

In the realm of molecular spectroscopy, the association between chemical bond lengths and the wavelength of absorbed radiation is crucial, particularly when studying electronic transitions. This relationship is underpinned by the principles of quantum mechanics, where molecular energy levels are quantised, and transitions between these levels correspond to the absorption or emission of electromagnetic radiation. Both chemical and mechanical treatments significantly influence the photoacoustic signal in cellulose fibres. HCl vapor treatment of cellulose induces complex, non-linear chemical modifications that profoundly affect its light-absorbing properties, leading to significant alterations in its spectroscopic behaviour and optical characteristics. Mechanical treatment of cellulose fibres derived from hardwood and softwood diminishes their capacity to absorb incident light, whereas chemical treatment enhances the absorption capabilities of cotton cellulose fibres, as evidenced by increased discrete wavelet transform energy levels. These variations in integrated DWT coefficient energy levels reflect structural changes in the cellulose due to different treatment durations, involving the breakdown of glycosidic bonds, alterations in crystalline regions, and changes in molecular weight. Coifman wavelets, with their heightened sensitivity to changes in signal structure, effectively highlight these modifications. The observed ‘increasing–decreasing’ pattern of energy can indicate the dynamics of the treatments and non-linear responses within the cellulose matrix. This comprehensive analysis not only advances our understanding of fibre modification processes but also enhances the potential for optimizing industrial applications of cellulose-based materials. Our findings pave the way for the development of tailored fibre treatments designed to improve material performance across various applications.

## Figures and Tables

**Table 1:** Exhibited peak locations on the wavelet spectra (nm).

**WFP, SW, HW wavelet PAS:**											
250.9	252.2	254.7	259.8	269.9	290.3	330.8					
**WFP wavelet RDB-PAS:**											
250.5	251.2	251.4	251.9	252.4	252.7	253.4	255.5	260.9	271.8	293.75	337.5
**HW wavelet RDB-PAS:**											
250.7		251.4	251.9	252.4	252.7	253.4	255.5	260.9	271.8	293.75	337.5
**SW wavelet RDB-PAS:**											
250.7		251.4	251.9	252.4	252.7	253.4	255.5	260.9	271.9	293.75	337.5

## Data Availability

The original contributions presented in the study are included in the article/Supplementary Materials, further inquiries can be directed to the corresponding author.

## Supplementary Materials

The following supporting information can be downloaded at https://www.mdpi.com/article/10.3390/polym16233419/s1, Figure S1: Schematic illustration of photoacoustic spectroscopy (PAS; left) and reversed double-beam photoacoustic spectroscopy (RDB-PAS). In practice, two beams are combined by a fiber combiner (Schott Moritex Quartz UV Light Guide MWS5-1000S-UV3) to be introduced in a PA cell; Figure S2: An example of results of RDB-PAS and PAS measurements. Left (top): RDB-PA spectrum, left (bottom): PA spectrum and right: ERDT pattern of sample WEP_6h; Refs. [4,15,40,41,42,43] are mentioned in Supplementary Materials.
