# Supplementary material for: Exploring the Molecular Structure and Treatment Dynamics of Cellulose Fibres with Photoacoustic and Reversed Double-Beam Spectroscopy
Source: Polymers (Basel). 2024 Dec 5;16(23):3419. doi: 10.3390/polym16233419 (PMC11644499; doi:10.3390/polym16233419)
Supplement: Supplementary file 1 [file polymers-16-03419-s001.zip › polymers-3299777-supplementary.pdf]

Supplementary Information

Exploring the molecular structure and treatment dynamics of cellulose fibres with photoacoustic and reversed double-beam spectroscopy

Levente Csóka[1,2*], Worakan Csoka[2], Ella Tirronen[2], Ekaterina Nikolskaya[2], Yrjö Hiltunen[2], Bunsho Ohtani[3]

Polymers Journal

[1]ELTE Eötvös Loránd University, Faculty of Informatics, 1053 Budapest, Hungary

[2]South-Eastern Finland University of Applied Sciences, Fiber Laboratory, 57200 Savonlinna, Finland

[3]Nonprofitable Organization touche NPO, 1-6-414, Norh 4, West 14, Sapporo 060-0004, Japan

Corresponding-author email: csl@inf.elte.hu (Levente Csoka)

Photoacoustic spectroscopy and reversed double-beam photoacoustic spectroscopy

A schematic illustration of photoacoustic spectroscopy (PAS) and reversed double-beam photoacoustic spectroscopy (RDB-PAS) is shown in **Fig. S1**. In PAS, conventional spectroscopy involves detecting the photoabsorption of a sample as sound, which is collected by a microphone. This is achieved by wavelength scanning of intensity-modulated light (e.g., LED) or chopped light (e.g., from a spectrometer). The observed signal (sound) intensity, assumed to be proportional to photoabsorption, is calibrated using the signal from a black, highly photoabsorbing material such as graphite to obtain a photoabsorption spectrum on a relative scale [1]. Although absolute photoabsorption cannot be determined in ordinary PAS measurements, PAS can measure photoabsorption without the interference of light scattering and reflection by the sample. Thus, even non-transparent solids, such as powders, can be measured. In this study, the photoabsorption-edge wavelength (CBB) was measured using PAS.

Reversed double-beam photoacoustic spectroscopy (RDB-PAS) is a newly developed variant of photoacoustic spectroscopy [2,3]. In RDB-PAS measurements, two beams are directed onto a sample within a photoacoustic (PA) cell equipped with a microphone. One beam is intensity-modulated LED light (typically 625 nm or 940 nm) that generates a PA signal from the sample, while the other is a continuous, wavelength-scanned light beam that induces a photoreaction in the sample without generating a PA signal at the wavelength of photoabsorption. The

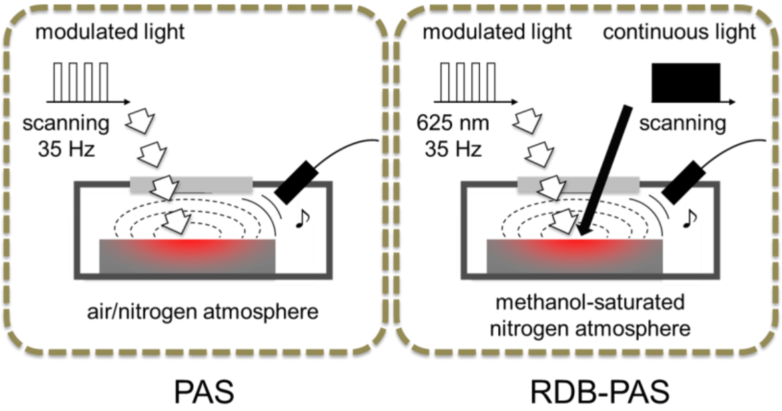

Figure S1 Schematic illustration of photoacoustic spectroscopy (PAS; left) and reversed double-beam photoacoustic spectroscopy (RDB-PAS). In practice, two beams are combined by a fiber combiner (Schott Moritex Quartz UV Light Guide MWS5-1000S-UV3) to be introduced in a PA cell.

resulting RDB-PAS spectrum is an action spectrum that reflects changes in photoabsorption at the modulated-light wavelength due to the photoreaction.

The term "reversed" in RDB-PAS refers to the different scanning arrangements of the two beams compared to traditional double-beam photoacoustic spectroscopy (DB-PAS). In RDB-PAS, the continuous beam's wavelength is scanned while the modulated beam's wavelength remains fixed. In contrast, DB-PAS involves scanning the modulated beam's wavelength while the continuous beam's wavelength remains constant [4]. Thus, while DB-PAS extends PAS to observe changes in photoabsorption spectra due to photoirradiation, RDB-PAS provides a distinct action spectrum based on these changes.

Energy-resolved distribution of electron traps

RDB-PAS was originally developed to measure the energy-resolved distribution of electron traps (ERDT) in solid materials. Electron traps (ETs) are essentially assumed vacant electronic states within solids. For example, metal oxides like titania (TiO_2) and ceria (CeO_2) turn grey or brown when heated in a vacuum or irradiated under a reductive atmosphere due to the reduction of cations, which results in the formation of low-valent metal ions [4,5]. The density of these reduced sites in titania can be quantified through photochemical titration using methyl viologen, revealing that the density is specific to the type of titania sample. These reduced sites, which trap electrons, are referred to as ETs [2,3].

For a range of metal-oxide powders, ETs can be filled with electrons by directly exciting electrons from the valence band into the ETs via photoirradiation, which results in an increase in photoabsorption. Thus, ERDT can be measured using RDB-PAS by scanning the wavelength of continuous light from longer to shorter wavelengths (lower to higher energy) to populate the ETs, while the accumulation of electrons is detected by a fixed-wavelength modulated light [2,3]. To prevent the de-excitation of electrons trapped in ETs, RDB-PAS measurements are conducted in a PA cell under a methanol-saturated nitrogen atmosphere. The methanol adsorbed on the sample surface may irreversibly capture positive holes, thereby stabilizing the trapped electrons.

For the cellulose used in this study, there has been no evidence suggesting the presence of electron traps (ETs). However, it has been reported that energy-resolved distribution of electron traps (ERDT) patterns can be obtained for cellulose samples [6] using a 940-nm LED for the modulated light beam. In contrast, measurements of titania samples using both 625-nm and 940-nm LEDs yielded almost identical results. This indicates that significant wavelength-dependent photoreactions occur in cellulose samples, leading to visible-light photoabsorption. Nevertheless, interpreting the obtained ERDT patterns for cellulose may be challenging due to the ambiguity regarding the actual structure of the species or sites identified as ETs, as discussed in this article.

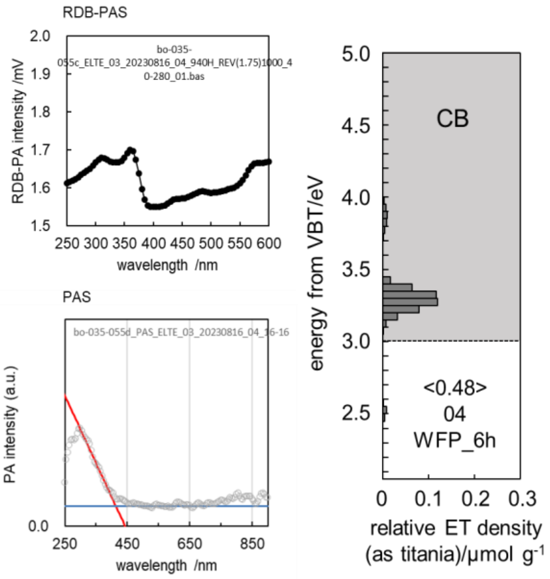

Figure S2 An example of results of RDB-PAS and PAS measurements. Left (top): RDB-PA spectrum, left (bottom): PA spectrum and right: ERDT pattern of sample WEP_6h.

Data analysis of ERDT patterns for cellulose samples

Fig. S2 illustrates an example of data analysis used to obtain an ERDT (energy-resolved distribution of electron traps) pattern for a cellulose sample (WFP_6h). The RDB spectrum (top left) indicates that the signal intensity is significantly lower compared to metal oxide powder samples. This low intensity could be due to (i) negligible electron traps (ETs) in the cellulose samples and/or (ii) a low photoabsorption coefficient of ETs at the detection wavelength of 940 nm.

In RDB-PAS measurements, fluctuations in PA signal intensity can occur due to temperature changes at the sample surface, as the PA signal is sensitive to temperature [1]. Although the cell temperature was controlled at 298.0 K using a block heating-cooling bath, the actual temperature of the sample surface may have varied, potentially causing the gradual decrease observed in the RDB-PA spectrum.

The RDB-PA spectrum reflects the total amount of filled ETs. Differentiating the RDB-PA spectrum from the higher-wavelength (lower-energy) side reveals the energy-resolved distribution of ETs, as shown on the right side of **Fig. S2**. For metal-oxide samples, ETs are filled by exciting electrons from the valence band, and their energy is referenced to the top of the valence band (VBT). However, for cellulose samples, the energy structure of such bands appears unusual.

In the PA spectrum (bottom left), photoabsorption increases below 450 nm. A linear fit was used to determine the photoabsorption edge energy (wavelength), which is indicated as a dotted line in the ERDT pattern.

References

[1] B. Ohtani: Photoacoustic Spectroscopy, Springer Handbook of Inorganic Photochemistry 303-313 (2022). [10.1007/978-3-030-63713-2]

[2] A. Nitta, M. Takase, M. Takashima, N. Murakami, B. Ohtani: A Fingerprint of Metal-oxide Powders: Energy-resolved Distribution of Electron Traps, Chem. Commun. **52**(81) 12096-12099 (2016). [10.1039/C6CC04999K]

[3] A. Nitta, M. Takashima, N. Murakami, M. Takase, B. Ohtani: Reversed double-beam photoacoustic spectroscopy of metal-oxide powders for estimation of their energy-resolved distribution of electron traps and electronic-band structure, Electrochim. Acta **264** 83-90 (2018). [10.1016/j.electacta.2017.12.160]

[4] N. Murakami, O.-O. Prieto-Mahaney, R. Abe, T. Torimoto, B. Ohtani: Double-Beam Photoacoustic Spectroscopic Studies on Transient Absorption of Titanium(IV) Oxide Photocatalyst Powders, J. Phys. Chem. C **111**(32) 11927-11935 (2007). [10.1021/jp071362x]

[5] S. Ikeda, N. Sugiyama, S.-y. Murakami, H. Kominami, Y. Kera, H. Noguchi, K. Uosaki, T. Torimoto, B. Ohtani: Quantitative Analysis of Defective Sites in Titanium(IV) Oxide Photocatalyst Powders, Phys. Chem. Chem. Phys. **5**(4) 778-783 (2003). [10.1039/b206594k]

[6] L. Csoka, W. Hosakun, O. Kolonics, B. Ohtani: Reversed double-beam photoacoustic spectroscopic analysis of photoinduced change in absorption of cellulose fibres, Sci. Rep. **12** 12475 (2022). [10.1038/s41598-022-18749-w]
